# Feasibility of Using a Luminescence-Based Method to Determine Serum Bactericidal Activity against *Neisseria gonorrhoeae*

**DOI:** 10.3390/vaccines7040191

**Published:** 2019-11-21

**Authors:** Fiona Clow, Conor J O’Hanlon, Myron Christodoulides, Fiona J Radcliff

**Affiliations:** 1 Department of Molecular Medicine and Pathology, Faculty of Medical and Health Sciences, University of Auckland, Auckland 1023, New Zealand; f.clow@auckland.ac.nz (F.C.); c.ohanlon@auckland.ac.nz (C.J.O.); 2 Faculty of Medicine, Academic Unit of Clinical and Experimental Sciences, Sir Henry Wellcome Laboratories, University of Southampton, Southampton SO166YD, UK; M.Christodoulides@soton.ac.uk

**Keywords:** *Neisseria gonorrhoeae*, serum bactericidal activity, luminescent, ATP, serum-sensitivity

## Abstract

Development of a vaccine to limit the impact of antibiotic resistant *Neisseria gonorrhoeae* is now a global priority. Serum bactericidal antibody (SBA) is a possible indicator of protective immunity to *N. gonorrhoeae*, but conventional assays measure colony forming units (CFU), which is time-consuming. A luminescent assay that quantifies ATP as a surrogate measure of bacterial viability was tested on *N. gonorrhoeae* strains FA1090, MS11 and P9-17 and compared to CFU-based readouts. There was a linear relationship between CFU and ATP levels for all three strains (*r* > 0.9). Normal human serum (NHS) is a common source of complement for SBA assays, but needs to be screened for non-specific bactericidal activity. NHS from 10 individuals were used for serum sensitivity assays—sensitivity values were significantly reduced with the ATP method for FA1090 (5/10, *p* < 0.05) and MS11 (10/10, *p* < 0.05), whereas P9-17 data were comparable for all donors. Our results suggest that measuring ATP underestimates serum sensitivity of *N. gonorrhoeae* and that the CFU method is a better approach. However, mouse anti-P9-17 outer membrane vesicles (OMV) SBA titres to P9-17 were comparable with both methods (*r* = 0.97), suggesting this assay can be used to rapidly screen sera for bactericidal antibodies to gonococci.

## 1. Introduction

Gonorrhoea is a sexually transmitted disease estimated to cause at least 78 million new cases worldwide per year [1]. The causal organism, *Neisseria gonorrhoeae,* is listed as a high priority pathogen for research into novel treatments by the WHO [2] due to its ability to rapidly develop resistance to antibiotics [3]. Isolates with resistance to the last recommended treatment combining ceftriaxone with azithromycin have been detected in several individuals from the United Kingdom [4,5] and Australia [6] shortly after overseas travel, emphasising the potential for global spread of intractable or even incurable *N. gonorrhoeae.* Infections are typically self-limiting and restricted to mucosal sites, however, untreated infection of females can lead to pelvic inflammatory disease, infertility and ectopic pregnancies [7]. Sexually transmitted infections including *N. gonorrhoeae* have also been linked with a significantly greater risk of contracting HIV [8]. 

Infection with *N. gonorrhoeae* results in an initial neutrophilic inflammatory response at the site of infection [9,10] and limited, short-lived humoral responses [11]. Experimental infection of human subjects [12] and longitudinal studies of high risk individuals [13,14] show that acquisition and subsequent clearance of an infection does not protect against further infections with *N. gonorrhoeae*. Humoral responses to highly variable but abundant cell wall components such as lipooligosaccharides (LOS) and pili, as well as cellular responses that are skewed towards T helper (Th) 17 and regulatory T (T_reg_) cells, are reported to be elevated after recent infection (reviewed in [15]). Recent studies in mice suggest that skewing mucosal responses away from Th17/T_reg_ axis towards Th1/cellular immunity leads to improved clearance of *N. gonorrhoeae* [16,17]. Multiple pre-clinical vaccine candidate antigens have been identified as having promising activity in mouse models (reviewed in [18]); of note, a peptide mimic that targets a highly conserved epitope of LOS shows particular potential but has not yet been tested in humans (reviewed in [19]). To date, those vaccines tested in humans have largely failed to stimulate protection from infection [20,21]. 

Detection of serum bactericidal antibody (SBA) is a key correlate of vaccine-induced protective immunity for *N. meningitidis* [22], a close relative of *N. gonorrhoeae* [23], and considered likely to be important for protection against *N. gonorrhoeae*. The SBA assay measures killing of bacteria after exposure to an exogenous source of complement and defined dilutions of immune antibodies, which leads to cell lysis by activation of the classical complement pathway [24]. Normal human serum (NHS) is often used in SBA assays as a source of complement, and a key component of assay validation is confirmation that addition of NHS alone does not initiate killing of bacteria. This is particularly important because *N. gonorroheae* clinical isolates show variable sensitivity to NHS, with those strains associated with disseminated disease more likely to be serum-resistant [25,26], as are those freshly isolated from clinical specimens [27]. The conventional methodology for detection of SBA or serum sensitivity of gonococci or meningococci uses enumeration by colony counting, which is labour intensive, requires an overnight incubation step and relies on large quantities of agar plates [28]. Use of a commercially available reagent for detecting bacterial ATP in microtitre plates using a luminescent readout as a surrogate for bacterial viability/colony forming units (CFU) has been described for measurement of SBA to *N. meningitidis* serogroups A and W, as well as several other pathogenic bacteria [29,30]. We describe the utility of this approach to measure serum sensitivity and SBA to gonococci.

## 2. Materials and Methods 

### 2.1. Culture of Bacteria

*N. gonorrhoeae* strains FA1090 (ATCC 700825), MS11 (ATCC BAA-1833) and P9-17 [27] were used in this study. Fresh cultures of bacteria were prepared from frozen stocks by streaking onto gonococcal agar (GCA) consisting of gonococcal (GC) agar base supplemented with 5 g/L bovine dried haemoglobin and 1% v/v IsoVitaleX (BD Biosciences, Franklin Lakes, NJ, USA). Bacteria were grown overnight at 37 °C, 5% CO_2_, then streaked onto fresh GCA and cultured for 6 h to produce mid-log cultures. In experiments using cytidine-5′-monophospho-N-acetylneuraminic acid (CMP-NANA, Sigma C8271), bacteria from overnight cultures were used to inoculate GC-proteose peptone broth (GCB) supplemented with 1% v/v IsoVitaleX with/without 4 µg/mL CMP-NANA [31] and incubated at 37 °C with shaking for approximately 3 h until an A_600_ of 0.45 (~2 × 10^8^ CFU/mL) was reached. 

### 2.2. Human and Murine Sera

Normal human serum (NHS) from healthy human donors was used as a source of complement for serum sensitivity and SBA assays. Peripheral blood was obtained by venipuncture under The University of Auckland Human Participants Ethics Committee approval, reference 021200. All donors gave informed written consent prior to blood sampling. Blood was allowed to clot for a maximum of 30 min, serum collected after centrifugation (1250 × g, 20 min, 4 °C) and aliquots stored at −80 °C. NHS was diluted to 16.7% v/v for use as a complement source, which is within the recommended range of 10%–20% v/v for gonococcal bactericidal assays [32]. NHS was heat-inactivated at 56 °C for 30 min before use (hiNHS) as a complement inactivated control. 

Anti-sera to gonococcal outer membrane vesicles (OMV) were raised in mice. Preparation of detergent-extracted *N. gonorroheae* OMV extracts from P9-17, FA1090 and MS11 was on the basis of the methodology described for isolation of *N. meningitidis* OMVs for human vaccination [33], with minor adaptations [34]. *N. gonorrhoeae* was grown overnight in GCB at 37 °C, 5% CO_2_ with gentle shaking, followed by incubation at 40 °C for 2 h. Briefly, bacteria were harvested by centrifugation and the pellet treated twice for 30 min with 0.1 M Tris-HCl pH 8.6, 10 mM EDTA and 0.5% w/v sodium deoxycholate. The supernatants underwent ultracentrifugation (100,000 × g, 2 h, 4 °C), then the pelleted material was suspended in PBS, filter sterilized and stored at 4 °C. The protein content of the OMV extracts was quantified with a Pierce BCA Protein Assay Kit (Thermo Fisher Scientific, Auckland, New Zealand). 

Groups of three female specific pathogen free CD1 mice aged 5–6 weeks were immunised via the intra-peritoneal route with 4 µg of *N. gonorrhoeae* OMV extract adsorbed to alum adjuvant (Alu-Gel-S, 2%, Serva) on days 0, 14 and 28. Baseline blood samples were collected prior to vaccination and immune serum was collected 7 days after the final immunisation. Blood was collected into microvette 500 Z-gel tubes (Sarstedt), and serum was harvested after 1 hour and stored at −80 °C. Immune sera were pooled for the SBA and were heat-inactivated before use as a source of anti-gonococcal immune antibodies in the SBA assay. 

Animal work was approved by The University of Auckland Animal Ethics Committee (protocol 1816) and was conducted in accordance with the university’s Code of Ethical Conduct and the Animal Welfare Act 1999. Mice were sourced from the Vernon Jansen Unit (University of Auckland, Auckland, New Zealand), monitored daily by qualified staff and suffered no adverse effects from these manipulations.

### 2.3. Comparison of CFU and ATP Readouts

*N. gonorrhoeae* was grown to mid-log on GCA and suspended in Dulbecco’s modification of PBS (PBSB) at a UV A_260_ of 1 (~4.2 × 10^9^ CFU/mL), further diluted to 4 × 10^8^ CFU/mL, then serial diluted in PBSB and incubated for 1 h at 37 °C, 5% CO_2_ in round-bottomed microtitre plates before measurement of ATP by luminescence or determination of CFU. The CFU of the starting inoculum was to confirm that bacteria numbers remained unaltered after 1 h in PBSB. For enumeration of colonies, 15 µL of the initial suspension was diluted, spread onto GCA without haemoglobin in triplicate using a sterile loop and incubated overnight at 37 °C, 5% CO_2_. Quantification of bacteria by measurement of ATP was determined with BacTiter-Glo (Promega) prepared as per the manufacturer’s instructions. After the 1 h incubation step, the microtitre plate was centrifuged at RT for 10 min at 3220 × g, supernatant was removed from each well and the pelleted bacteria was re-suspended in PBSB. Half of this suspension was transferred to a 96-well white flat bottom microtitre plate (Greiner Bio-One, Kremsmünster, Austria) and an equal volume of BacTiter-Glo reagent added to each well. The reaction was incubated for 5–10 min at RT on an orbital shaker before the luminescence signal was read using a Victor X Light 2030 Luminescence Reader (PerkinElmer, Waltham, MA, USA). Each plate was read three consecutive times and the average counts per second (CPS) for each reaction recorded. The background signal from a blank well containing PBSB was subtracted from each reading.

### 2.4. Measurement of Serum Sensitivity

*N. gonorrhoeae* was grown to mid-log on GCA and suspended in Dulbecco’s modification of PBS (PBSB) as detailed above. Reaction mixes containing ~1 × 10^3^ CFU *N. gonorrhoeae* and 16.7% v/v NHS or heat-inactivated NHS control for every donor were adjusted to a final volume of 0.1 mL in PBSB-hiFBS (PBSB-1% v/v heat-inactivated foetal bovine serum (Thermo Scientific, Waltham, MA, USA)). The reaction mix was incubated for 1 h at 37 °C, 5% CO_2_ in round-bottomed microtitre plates and bacteria enumerated as per Section 2.3. Reaction mixes for ATP-based measurement of bacterial killing were identical to the CFU-based method, except that ~5 × 10^3^ CFU bacteria were used in the reaction mix for this assay; downstream processing was as per Section 2.3. Serum sensitivity was calculated as a percentage of CFU or CPS signal in the presence of NHS compared to hiNHS. Values were reported as zero when bacteria numbers increased in the presence of NHS.

### 2.5. Quantification of SBA

Measurement of SBA by CFU was determined as previously described [35], with minor modifications for quantification of bacterial ATP by luminescence. *N. gonorrhoeae* was grown to mid-log on GCA and suspended in Dulbecco’s modification of PBS (PBSB). Reaction mixes containing ~1 × 10^3^ CFU *N. gonorrhoeae* (CFU) or ~5 × 10^3^ CFU (ATP), 16.7% v/v NHS and 10% v/v diluted heat-inactivated murine anti-sera were adjusted to a final volume of 0.1 ml in PBSB-hiFBS. The murine anti-sera was diluted to a starting titre of either 1/16 or 1/32, then diluted twofold in PBSB to 1/2048. NHS- and hiNHS-only assay control reactions were included in every assay. The reaction mix was incubated for 1 hour at 37 °C, 5% CO_2_ in round-bottomed microtitre plates, and bacteria were enumerated as per Section 2.3. Bacterial survival was calculated by expressing test CFU or CPS values as a proportion of the maximum CFU or CPS value (i.e., the NHS-only reaction). Survival values calculated as >100% were reported as 100%. The titre was defined as the reciprocal of the interpolated serum dilution that killed 50% of the bacteria in comparison to the NHS only reaction. Titres were log-transformed for plotting and determination of exact SBA titres. These were calculated using Opsotiter3, an Excel-based data processing program from The University of Alabama at Birmingham reference library (UABRF) and licenced from UABRF [36,37,38].

### 2.6. Statistical Analysis

All data were from three independent biological repeats. The results from each biological repeat are either shown individually on a single plot or combined to display mean ± standard deviation. All statistical analyses were carried out in GraphPad Prism Version 7.03 (GraphPad Software, Inc., San Diego, CA, USA); the tests applied are indicated in the figure legends.

## 3. Results

### 3.1. Comparison of Luminescent and CFU Readouts

**Figure 1:**
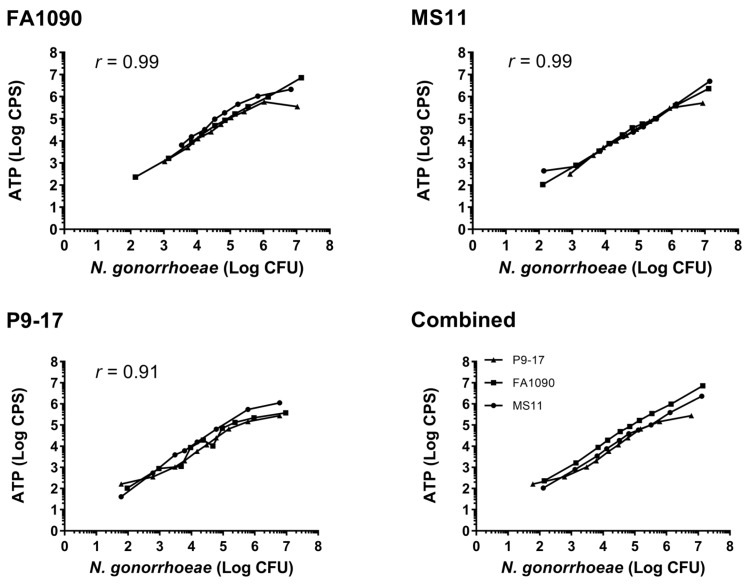
Quantification of *Neisseria gonorrhoeae* by detection of colony forming units (CFU) versus ATP. Results show mean values from three independent assays, presented either individually for each strain or as combined mean values from all three strains. The correlation between CFU and ATP was calculated for each strain using a two-tailed Pearson coefficient (*p* < 0.001 for each strain).

To determine whether BacTiter-Glo is a viable choice to quantify *N. gonorrhoeae* survival, initial experiments compared ATP readings from three *N. gonorrhoeae* isolates across a broad range of CFU (~200–10^7^ CFU/well). The ATP (luminescent) signal rose with increased *N. gonorrhoeae* CFU across the range tested (Figure 1). There was a high correlation between CFU and ATP levels for all strains across the full range of concentrations tested (*r* > 0.9), with the most reliable readouts in the range of 2 × 10^3^–1 × 10^6^ CFU/well for each of the three *N. gonorrhoeae* strains tested (Figure 1). All subsequent experiments used ~5 × 10^3^ CFU bacteria/well for the ATP assay to ensure a strong signal above background.

### 3.2. Use of Bioluminescent Assay to Screen for NHS Sensitivity

**Figure 2:**
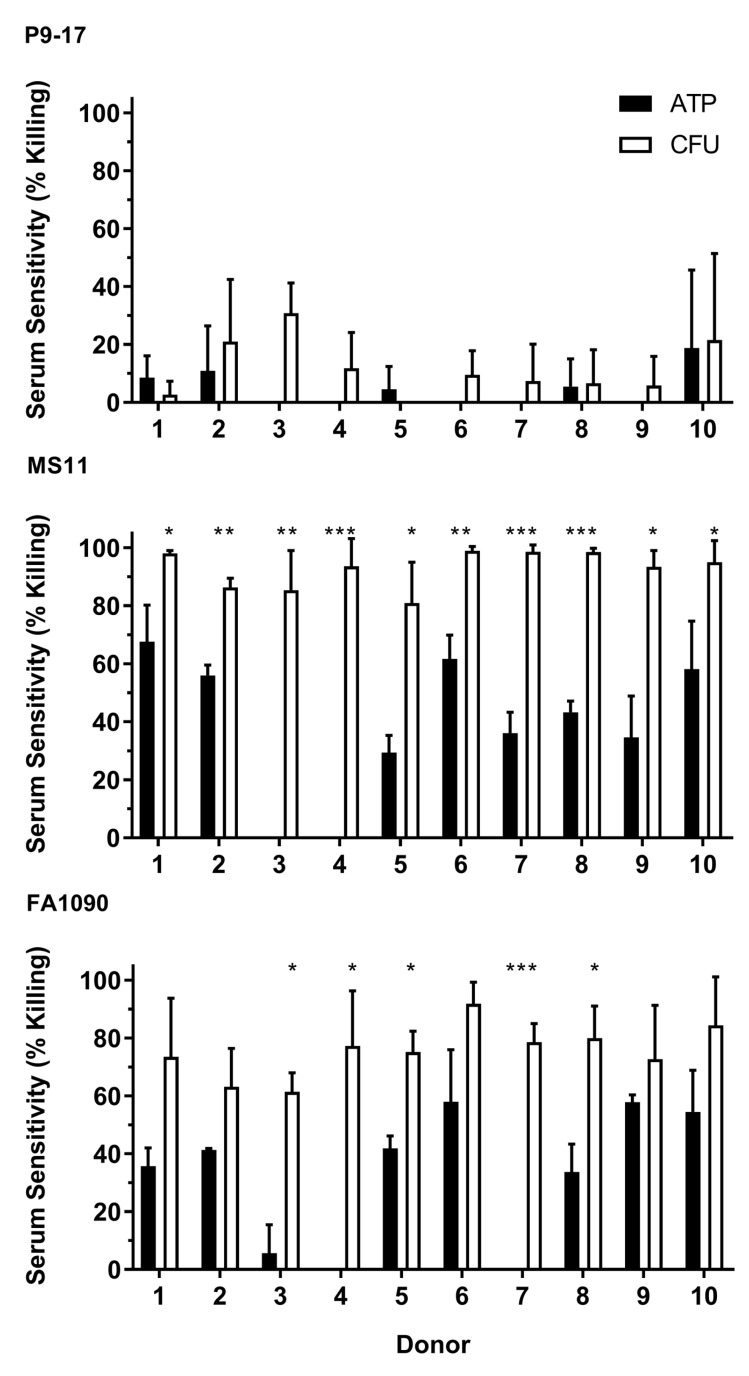
Detection of serum sensitivity of *N. gonorrohoeae* by enumeration of CFU versus ATP. *N. gonorrhoeae* strains P9-17, MS11 and FA1090 were tested for sensitivity to serum from 10 healthy human donors and samples taken to detect bacterial ATP or CFU. Data are combined means + SD from three independent experiments. Statistically significant differences in sensitivity between the two methods were determined using multiple *t*-tests with the Holm–Šidák method applied. * *p* < 0.05; ** *p* < 0.01; *** *p* < 0.001.

Sera from 10 NHS donors were screened for bactericidal activity against *N. gonorrhoeae* P9-17, MS11 and FA1090 using both CFU enumeration and ATP readouts in parallel. Reported levels of bacterial killing were either lower or equivalent in the ATP assay relative to enumeration of CFU in all three strains (Figure 2). The P9-17 strain had low levels of serum-mediated killing (0%–30%) after exposure to sera from all 10 donors and both methods produced similar results (Figure 2). In contrast, serum sensitivity results from the two assays diverged significantly for the MS11 strain (*p* < 0.05 for all donors) and the FA1090 strain (*p* < 0.05 for 5/10 donors). Of particular note, serum from donors 3 and 4 elicited little or no bactericidal activity in the ATP assay, but between 60%–90% killing after enumeration of CFU from both *N. gonorrhoeae* FA1090 and MS11. On the basis of these results, the P9-17 and MS11 strains had the best profiles for use in ATP-based SBA assays, representing resistant and susceptible strains, respectively. Unless specified otherwise, serum from donor 2 was used as a source of complement for all subsequent assays, in combination with the *N. gonorrhoeae* P9-17 strain.

### 3.3. Quantification of SBA by Detection of ATP

**Figure 3:**
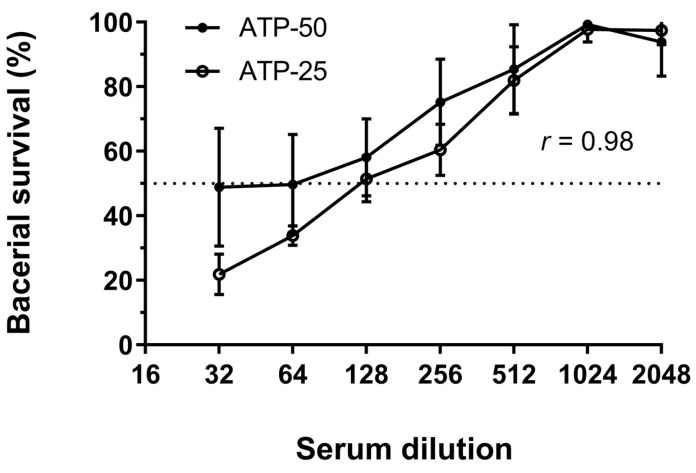
Comparison of two different resuspension volumes for quantification of serum bactericidal antibody (SBA) by ATP. Pooled anti-sera from mice immunized with *N. gonorrhoeae* P9-17 OMVs was tested for SBA to *N. gonorrhoeae* P9-17. Data are combined means ± SD from three independent experiments with 50 µL (ATP-50) or 25 µL (ATP-25) of re-suspended bacteria used to quantify ATP levels. The correlation between the two volumes was calculated using a two-tailed Pearson coefficient (*p* < 0.0001) and significant differences between paired sets of values were determined by multiple *t*-tests.

Assays to determine the SBA of murine anti-sera raised to *N. gonorrhoeae* P9-17 OMV extracts against *N. gonorrhoeae* P9-17 were carried out using the CFU or ATP method in parallel to directly compare the outcome. Each individual test used the same stock of bacteria and reaction mix. Bacteria were re-suspended in a volume of 100 µL buffer and 50 µL of this suspension used for detection of ATP in initial feasibility tests for this assay (Figure 1). However further testing showed that comparable results were obtained from bacteria re-suspended in 30 µL buffer, with 25 µL of this suspension used for detection of ATP (Figure 3). This adjustment meant that a larger proportion of bacteria from the reaction mix were used for reading out luminescence (>80% vs. 50%) and yielded similar results (*r* = 0.98). There were no significant differences between any paired sets of data, but standard deviations were reduced and detection of bacterial viability at serum dilutions of 1/32 and 1/64 was improved with the smaller re-suspension volume. This modification reduced the use of a costly reagent without affecting the data obtained, and therefore was adopted for all further assays. 

**Figure 4:**
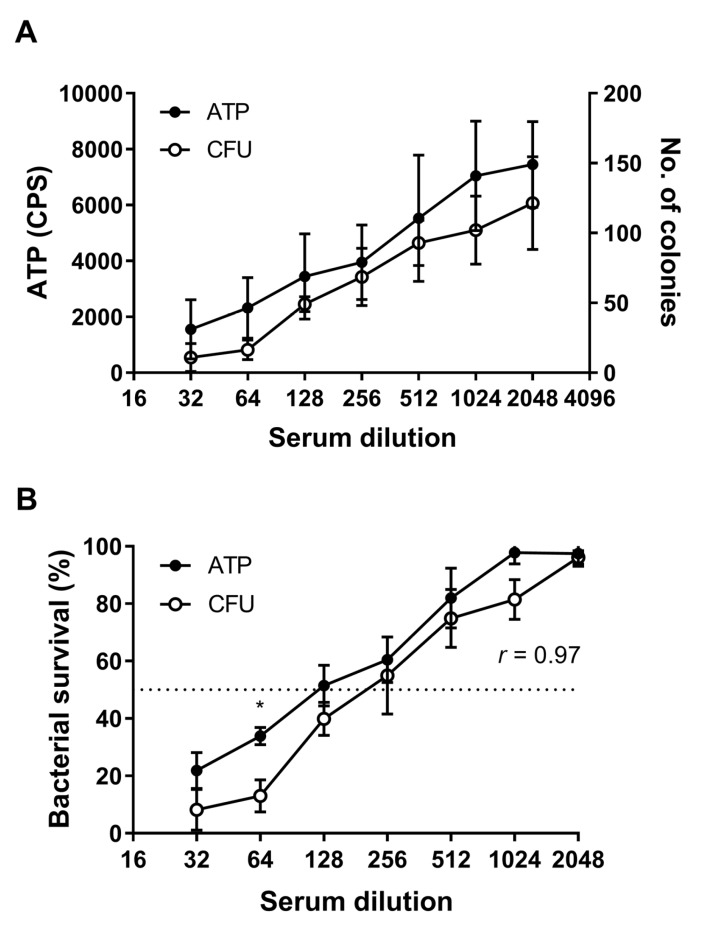
Comparison of SBA data obtained using CFU or ATP methods to quantify bacteria. Raw ATP (luminescence) values and colony counts obtained in an assay for SBA (**A**) and a comparison of SBA titres using CFU and ATP methods (**B**). Serial twofold dilutions of murine serum raised to OMV extracts from *N. gonorrhoeae* P9-17 were incubated with live *N. gonorrohoeae* P9-17. Data are combined means ± SD from three independent experiments. ATP values were scaled to the left axis and CFU values to the right axis in (**A**). The correlation between CFU and ATP values was calculated using a two-tailed Pearson coefficient. Differences between matched sets of CFU or ATP values were determined using multiple *t*-tests. * *p* < 0.05.

A comparison of the range of raw values obtained with the ATP method versus colony counting readouts is shown in Figure 4A. Detection of ATP via luminescence gave readouts ranging from an average of 1552 CPS at a serum dilution of 1/32 through to 6756 at a dilution of 1/2048. Enumeration by colony count gave values of 11 to 126 CFU at the same dilutions. Control wells comprising bacteria incubated in NHS alone had average values of 6755 CPS and 126 CFU, respectively. There was a degree of variability in the range of raw values, particularly for the ATP assay (Figure 4A), but importantly readouts of bacterial survival remained consistent between assays (Figure 4B). The average SBA titre obtained with the CFU assay was higher than the ATP assay (223 vs. 134) and the average maximum killing was also increased in the CFU assay relative to the ATP assay (92% vs. 78%) (Figure 4B). However, a direct comparison of quantification of SBA titres by CFU and ATP showed a strong correlation between the two methods (*r* = 0.97, *p* < 0.001). Although bacterial survival was higher at all dilutions using the ATP method, a significant difference between the assays was only evident at 1/64, *p* < 0.05 (Figure 4B). The ATP assay was performed with approximately five-fold more bacteria, which may partially account for the trend towards a lower titre and high bacterial survival. 

**Figure 5:**
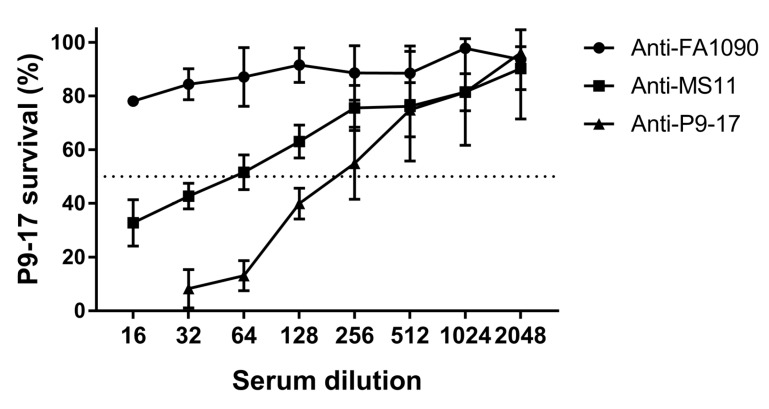
Measurement of cross-reactive SBA to *N. gonorrhoeae* P9-17 by detection of ATP. Serial twofold dilutions of murine sera raised to OMV extracts from *N. gonorrhoeae* P9-17, FA1090 or MS11 were incubated with *N. gonorrhoeae* P9-17 and bacterial survival measured by ATP (luminescence). Data are combined means ± SD from three independent experiments.

Antisera to OMV preparations from the other *N. gonorrhoeae* isolates were tested for cross-reactive SBA using the *N. gonorrhoeae* P9-17 ATP SBA assay; the anti-P9-17 OMV SBA data shown in Figure 4 were included as a point of comparison. A twofold titration starting at 1/16 showed no killing activity with sera against FA1090 OMVs (bacterial survival >80% at all dilutions tested), whereas anti-sera against MS11 OMVs had moderate cross-reactive bactericidal activity against *N. gonorrhoeae* P9-17 (endpoint titre = 56, maximum killing = 67%) (Figure 5). 

**Figure 6:**
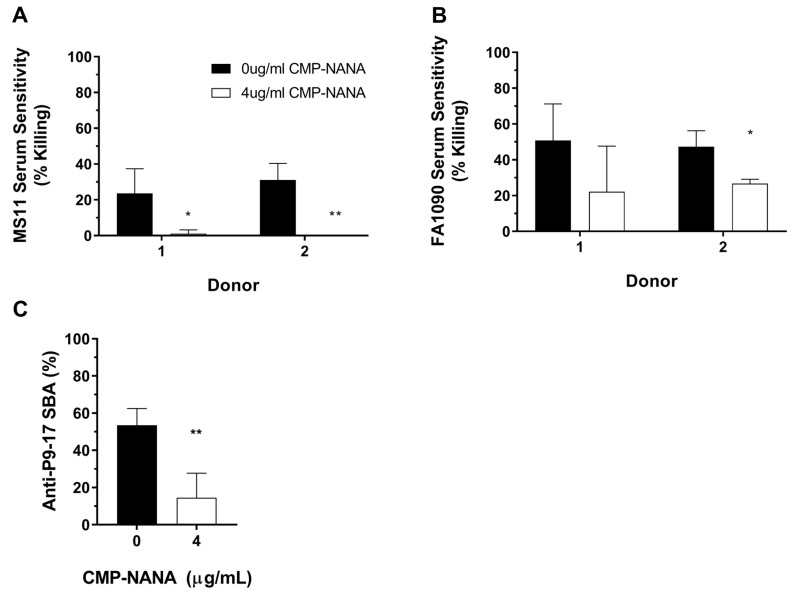
Reduction of serum or SBA sensitivity or after sialylation with cytidine-5′-monophospho-N-acetylneuraminic acid (CMP-NANA). Mid-log broth subcultures of *N. gonorrhoeae* MS11 (**A**) or FA1090 (**B**) were incubated with/without the addition of 4 μg/mL CMP-NANA and samples taken to detect bacterial ATP. The P9-17 strain (**C**) was incubated under the same conditions with the addition of murine anti-P9-17 OMV anti-sera at a dilution of 1/64 to examine the effect of sialylation on SBA. Data are combined means + SD from three independent experiments; statistically significant differences in sensitivity were determined using multiple *t*-tests with the Holm–Šidák method applied (**A**,**B**) or a paired *t*-test (**C**). Values were reported as zero in experiments where bacteria numbers had increased in the presence of NHS. * *p* < 0.05; ** *p* < 0.01.

Sialylation of lipooligosaccharides (LOS) is linked with the resistance of *N. gonorrhoeae* to serum-mediated killing by preventing activation of the complement cascade [31,39]; this is a critical variable to consider when determining potential bactericidal activity of anti-gonococcal immune sera. The FA1090 and MS11 strains, both of which showed a degree of sensitivity to sera from donors 1 and 2 (Figure 2), were chemically sialylated with 4 µg/mL CMP-NANA to verify that reduced bacterial killing could be detected using quantification of ATP as a readout. Treatment with CMP-NANA prevented killing of *N. gonorrhoeae* MS11 (Figure 6A; *p* < 0.05 for donor 1 and *p* < 0.01 for donor 2) and reduced NHS mediated killing of *N. gonorrhoeae* FA1090 by approximately 50% (Figure 6B; *p* = 0.203 for donor 1 and *p* < 0.05 for donor 2). A reduction in SBA activity after sialylation of the P9-17 strain was also detected using the ATP assay (53% vs. 14.5% killing at a serum dilution of 1/64, *p* < 0.01; Figure 6C).

## 4. Discussion

Detection and quantification of *N. gonorrhoeae* ATP using a commercially available luminescent substrate showed utility as an alternative approach to manual enumeration of CFU for measuring SBA to *N. gonorrhoeae*. In contrast, screening of NHS from multiple donors using the ATP method under-estimated serum sensitivity of the FA1090 and MS11 strains relative to the CFU method, suggesting that it is unsuited to this particular assay. These results indicate that detection of bacterial viability by ATP is most appropriate for assays where rapid killing of bacteria is expected, such as SBA, where killing is initiated by rapid activation of the classical complement pathway, leading to formation of C5b9 and lysis of gram-negative bacteria, a process that occurs within minutes of exposure [40]. To the best of our knowledge, this is the first report detailing the use of an ATP assay to measure SBA to *N. gonorrhoeae*. In line with an earlier report describing the utility of this reagent for other bacteria [29], use of this method to measure SBA offers multiple advantages, including a substantially reduced time to acquire results (3 h vs. >24 h), a sizeable decrease in hands-on time by laboratory personnel and the capacity to test large sets of samples in a single day. The cost of reagents and consumables for the ATP assay is lower than the conventional plate-based method. This assay could also be applied to other tests that rely on measuring *N. gonorrhoeae* viability, such as screening of antimicrobials, to streamline testing and identification of new drugs. 

The *N. gonorrhoeae* strains used in this study were chosen because they are frequently used for identification or testing of novel vaccine candidate antigens [35,41,42], for understanding gonococcal virulence [43] and for use as challenge strains for murine [44,45,46] or human models of infection [9,10]. Detection of bacteria using CFU versus ATP readouts produced a similar outcome with all three strains, suggesting that comparable results are likely to be obtained with other *N. gonorrhoeae* isolates. Studying clinically relevant strains will be important for determining potential strain-dependent variability of SBA in humans. The P9-17 strain was the most tractable strain in this study—it showed little or no serum sensitivity to NHS from multiple donors when measured by the CFU or ATP methods. In comparison the MS11 and FA1090 strains, both showed sensitivity to many or all of these sera under the conditions used, with higher levels of killing observed when bacterial survival was quantified by CFU. These results emphasise that NHS donors need to be screened carefully and/or that a smaller quantity of NHS should be used as a source of complement for assays using these strains. The FA1090 strain showed a surprising degree of serum susceptibility, given it is widely reported as a serum-resistant strain [10]. However, serum resistance can be lost with in vitro passaging [27,47], which is the most likely explanation for this unexpected result. Alternatively, a portion of our NHS donors may have cross-reactive bactericidal antibodies to some strains of *N. gonorrhoeae*. Of note, a large proportion of New Zealand’s population (~1 million) received an OMV-based vaccine to *N. meningitidis* serogroup B (MenB) from 2004 to 2008 [48], and some of these individuals may have entered our pool of NHS donors. The NHS used in this study was not pre-treated to eliminate cross-reactive antibodies [28], but is an important consideration for future studies. Alternatively, it was demonstrated that serum sensitivity of both these strains could be reduced by sialylation with CMP-NANA, with the caveat that this is likely to inhibit SBA mediated killing. Reduced susceptibility to SBA-mediated killing was shown with *N. gonorrhoeae* P9-17 sialylated with CMP-NANA relative to untreated bacteria.

Determination of bacterial survival by the ATP method was sufficient to show development of higher SBA titres to the P9-17 strain with anti-sera raised to homologous OMVs, compared to an intermediate or no detectable cross-reactive SBA with anti-sera against MS11 or FA1090 OMVs, respectively. The homologous anti-P9-17 SBA response reported here is similar to a previous study, which indicated that vaccination of mice with sodium deoxycholate-extracted OMVs from P9-17 stimulated a SBA titre of 256 when delivered in alum adjuvant, compared to a titre of >1000 with OMVs prepared without detergent [35]. 

Some limitations of the ATP approach were observed. Reliable quantification of *N. gonorrhoeae* required the preparation of a reaction mix containing a higher number of bacteria (~5 × 10^3^ CFU) to ensure luminescence readouts were well above background. This was partially because a portion of the re-suspended bacteria had to be transferred into another plate and combined with an equal volume of substrate for detection of ATP by luminescence. Results obtained with this higher number of bacteria were compared to a well-established CFU-based SBA that used ~1 × 10^3^ CFU [35]. The reduced serum sensitivity and antibody-mediated bacterial killing detected with the ATP assay was likely due to the addition of approximately five-fold more bacteria. Use of higher numbers of bacteria may have been responsible for the variation in serum-sensitivity between the ATP and CFU readouts, potentially by reducing or delaying non-specific serum mediated killing. Therefore, in its current format, the ATP assay under-estimates serum sensitivity of *N. gonorrhoeae* and should not be used in place of the traditional CFU-based method. However, despite the addition of higher numbers of bacteria for the ATP assay, detection of bacterial survival after incubation with bactericidal anti-sera was not significantly altered when compared to the conventional CFU-based method. Successful detection of SBA in sera from human subjects infected or recovering from infection with gonorrhoea has been reported using up to ~10^7^ CFU without a substantial loss of sensitivity [49]. 

Another significant difference between the standard CFU method and measurement of ATP is in processing time. *N. gonorrhoeae* and anti-sera are typically combined for 30–60 minutes prior to plating for CFU [28]. Plating for determination of CFU can be time-consuming, particularly if a large-scale assay is performed. Until transfer to agar plates and incubation, the bacteria remain in sub-optimal conditions for survival and continue to be exposed to the active components of the reaction. In contrast, the bacteria are pelleted and lysed for immediate quantification of ATP, a process that takes approximately 15 min, and therefore there is no potential for additional killing to occur. These differences in processing may partially explain the enhanced bacterial survival observed when serum sensitivity was compared using both ATP and CFU readouts. 

It has recently been observed that MenB vaccines reduce rates of gonorrhoea at a population level [50,51,52,53], which now provides the impetus to retrospectively examine historic resources, such as samples from trials with New Zealand’s MeNZB vaccine [54], as well as materials generated from more recent vaccine trials, to understand possible mechanisms of protection. This assay can be used to aid the global effort to develop a vaccine for gonorrhoea by facilitating testing of sera for possible cross-reactive SBA conferred by MenB OMV vaccines or for testing novel vaccine candidate antigens for bactericidal effects.

## 5. Conclusions

Measurement of *N. gonorrhoeae* survival by detection of ATP in a luminescent assay produced comparable results to manual plating and quantification of bacterial CFU for determination of SBA. Luminescent assays have the advantage of being high throughput, with a fast turnaround, offering the prospect of being able to screen large banks of samples for bactericidal activity relatively quickly. This approach could also be applied to contemporary clinical isolates of *N. gonorrhoeae*, including those with known anti-microbial resistance profiles, for rapid testing of new drugs. The development of new assays to study *N. gonorrhoeae* is a high priority because multi-drug resistant strains are a global concern and there is an urgent need to identify alternative treatments such as novel antibiotics or a vaccine.
